# *In vitro* and *in silico* insights on the regulation by gonadal hormones of pituitary GnRH receptor expression in a basal teleost, the European eel

**DOI:** 10.3389/fendo.2025.1673260

**Published:** 2025-09-29

**Authors:** Chien-Ju Lin, Karine Rousseau, Ching-Fong Chang, Sylvie Dufour

**Affiliations:** ^1^ Center of Excellence for the Oceans, National Taiwan Ocean University, Keelung, Taiwan; ^2^ Department of Aquaculture, National Pingtung University of Science and Technology, Pingtung, Taiwan; ^3^ Laboratory Molecular Physiology and Adaptation (PhyMA), National Museum of Natural History (MNHN), National Center of Scientific Research (CNRS), Paris, France; ^4^ Laboratory Biology of Aquatic Organisms and Ecosystems (BOREA), National Museum of Natural History (MNHN), Sorbonne Université, National Center of Scientific Research (CNRS), Institute of Research for Development (IRD), Paris, France

**Keywords:** GnRH receptor, promoter, sex steroids, activins, pituitary cells, *Anguilla*, teleosts, mammals

## Abstract

Eel species are basal teleosts with a unique life cycle including an arrest of sexual maturation before the reproductive oceanic migration. Our early studies showed that this blockade results from a deficient production of pituitary gonadotropins, due in part to a low responsiveness to gonadotropin-releasing hormone (GnRH). Three GnRH receptors have been identified in the eel, among them *gnrhr2* is the main pituitary receptor whose expression increases during the sexual maturation induced by gonadotropic treatments. We investigated the role of gonadal hormones in the feedback regulation of *gnrhr2* expression in the eel. The effects of steroids and activins were tested *in vitro* on primary cultures of eel pituitary cells and *gnrhr2* transcripts measured by qPCR. *In silico* analysis of eel *gnrhr2* promoter was performed to predict transcription factor binding sites and comparisons were made with *gnrhr* promoters from other teleosts and mammals. Estradiol and testosterone strongly and dose-dependently increased *gnrhr2* transcript levels as measured by qPCR. This stimulatory regulation was not observed with a non-aromatizable androgen, 11 keto-testosterone, and the effect of testosterone was abolished in the presence of an aromatase inhibitor, fadrozole, indicating an estrogen-specific positive control of eel *gnrhr2* expression. Other steroids, progesterone and cortisol, had no effect on *gnrhr2* expression. Gonadal peptides, activins A and B, were also tested, and showed an inhibitory effect on *gnrhr2* expression. Our results show that gonadal steroids exert a positive feedback, mediated by estradiol, on pituitary sensitivity to GnRH in the eel, in line with the regulatory mechanisms of the ovulatory luteinizing hormone (LH) surge in mammals. While investigation on *gnrhr* promoters is significantly lacking outside mammals, *in silico* analysis of the eel *gnrhr2* promoter allowed us to infer transcription factor binding sites potentially involved in the regulation of *gnrhr2* expression. Comparison was made with *gnrhr* promoters from other teleosts and mammals to discuss their evolutionary conservation. This study in the eel, a basal teleost representative, contributes to our understanding of the regulatory mechanisms of the complex eel life cycle and to raise basic knowledge on the regulation and evolution of pituitary GnRH receptivity in vertebrates.

## 1 Introduction

In vertebrates, gonadotropin-releasing hormone (GnRH) is the main brain actor controlling the reproductive gonadotropic axis (hypothalamus-pituitary-gonad axis) [for review (1)]. This neurohormone acts on the pituitary via specific G-protein coupled receptors, gonadotropin-releasing hormone receptors (GnRHR), to induce the synthesis and release of gonadotropins, luteinizing hormone (LH) and follicle-stimulating hormone (FSH). In some mammals, two receptor genes (*gnrhr1* and *gnrhr2*) have been characterized. However, in most mammalian species, no functional GnRHR2 is produced due to disruption of gene coding sequence and in the mouse *Mus musculus*, the *gnrhr2* gene is completely absent [for review (2)]. In mammals, GnRHR1 has been largely explored for its involvement in the regulation of pituitary gonadotropins, while GnRHR2 is thought to exert other functions at the brain and peripheral levels [for reviews (2, 3)]. For instance, recent studies in pigs, comparing *gnrhr2* knockdown line with control littermates, reported a potent direct action of GnRH2 on gonadal steroidogenesis and gametogenesis in both males and females, without affecting gonadotropins [for review (4)].

In 1997, de Roux and collaborators described the first mutations in the human GnRHR1 in a family with idiopathic hypogonadotropic hypogonadism, characterized by delayed puberty, low gonadotropin and sex steroid levels (5). During childhood, the gonadotropic axis is quiescent, involving a low responsiveness of pituitary gonadotroph cells to GnRH, which partly reflects a reduced expression of the GnRHR1 [for review (6)]. In mice, induced GnRHR1 mutations led to a phenotype similar to the clinical syndrome of hypogonadotropic hypogonadism (7, 8). Ontogeny of pituitary GnRHR1 in male and female rats revealed that its number or expression was maximal in the prepubertal period, when serum, pituitary content and expression levels of both gonadotropins are elevated (9–11).

A number of investigations in mammals reported positive and negative effects of sexual steroids on pituitary responsiveness to GnRH. Orchidectomy and ovariectomy in rats induce drastic increase in pituitary GnRHR number (12, 13) and mRNA levels (14), suggesting a negative feedback of gonadal hormones. Similarly, in castrated adult male Rhesus monkeys *Macaca mulatta*, an increase of pituitary *gnrhr* mRNA levels was observed (15). However, species-differences exist in mammals and in contrast to rats and Rhesus monkeys, gonadectomy in mice induces a decrease in GnRHR in both sexes indicating a positive feedback of gonadal steroids (16, 17). No effect of gonadectomy was reported on either the number (18) or the mRNA levels (19) of GnRHR in ewe.

In the female of mammalian species, including mouse, rat, ewe, cow and monkey, estradiol (E2) is considered a major positive regulator of preovulatory LH surge by increasing both pituitary sensitivity to GnRH and GnRH release [for review (20)]. During the estrous cycle, in rat, a positive correlation exists between circulating concentrations of E2 and GnRHR number (21, 22) and mRNA levels (14, 23). Similarly, in the ewe, maximal concentrations or mRNA levels of GnRHR are observed prior to LH surge when plasma E2 concentration are rising (24–26). Early studies demonstrated that E2 stimulates the expression and number of GnRHR by a direct pituitary effect as shown on primary cultures of pituitary cells in rat (27, 28), mouse (29) and sheep (20, 30) [for review (31)].

Activins are peptide hormones produced by the gonads, pituitary and other organs, which were first identified in porcine follicular fluid for their stimulatory role on FSH release by pituitary gonadotrophs (32). Beside their direct stimulatory role on FSH expression and release, activins were also shown to increase the synthesis of GnRHR by cultured rat pituitary cells (33) and the expression of *gnrhr* by the mouse gonadotroph cell line alphaT3 (34). This supports a role of activins in the pituitary sensitivity to GnRH [for review (35)].

In teleosts, the pioneering studies by Breton and collaborators (36) revealed the presence of a hypothalamic gonadotropin-releasing factor in common carp *Cyprinus carpio*, and Sherwood and colleagues (37) identified the GnRH sequence in chum salmon *Oncorhynchus keta*. Since then, teleost GnRH and GnRHR have been and still are the subject of extensive ongoing investigation [for review (38)]. While only one or two GnRHR are present in mammals, up to six *gnrhr* gene paralogs have been identified in some teleosts such as the Atlantic salmon (39). A recent phylogenetic analysis of vertebrate GnRHRs by Ciani and collaborators divides them into two main types, GnRHR1 and GnRHR2, each divided into further subtypes (39). At sexual maturation, an increase of the pituitary expression of *gnrhr* was reported in various teleost species, such as in European seabass *Dicentrarchus labrax* (40), Nile tilapia *Oreochromis niloticus* (41), pejerrey *Odontesthes bonariensis* (42), Atlantic cod *Gadus morhua* (43), Atlantic salmon (39, 44, 45), and chub mackerel *Scomber japonicus* (46). Our previous studies in the black porgy *Acanthopagrus schlegeli* showed a specific increase of a pituitary GnRH receptor (*gnrhr1*, corresponding to *gnrhr2bb* in Ciani’s nomenclature) during sexual maturation, as well as under *in vivo* treatments with human chorionic gonadotropin (hCG) or sex steroids (47). We also demonstrated a stimulatory effect of sex steroids on *gnrhr1 (gnrhr2bb)* expression by black porgy pituitary cells *in vitro* (47).

Among teleosts, *Anguilla* species are of special phylogenetic and biological interest. As members of the group of elopomorphs, they are extant representatives of basal teleosts. The eels, *Anguilla* genus, encompass about nineteen species and subspecies, distributed in the Indian, Pacific and Atlantic Oceans, and all possessing a peculiar life cycle with a reproduction in oceanic area and a juvenile growth in continental watersheds [for review (48)]. Future genitors, named silver eels, migrate downstream towards the ocean, but remain blocked at prepuberty as long as the oceanic migration is prevented [for review (49)]. Experimental sexual maturation of female and male silver eels can be induced by gonadotropic treatments, as first demonstrated by the work of Fontaine and coworkers in the European eel, *Anguilla anguilla* (50, 51). Such gonadotropic treatments, fish pituitary extract in the female and human chorionic gonadotropin in the male, are currently used to induce experimental maturation in various eel species, and the biological cycle has been successfully closed in the case of the Japanese eel, *A. japonica* [for review (52)]. As shown by our previous studies in the European eel, the prepubertal blockade is due to a deficiency in pituitary gonadotropin production, itself resulting from a dual brain control: firstly, a teleost species-specific strong inhibition by dopamine (DA); and secondly, similar to the situation in mammals before puberty, a lack of stimulation by GnRH, including both a low production of GnRH and a low pituitary sensitivity to GnRH [ (53); for review (54)]. A triple treatment with sex steroids (E2 or testosterone, T), GnRH agonist and DA antagonist is thus able to stimulate pituitary LH synthesis and release, and subsequent ovarian vitellogenesis (53, 55).

Three GnRH-R genes have been identified by Peñaranda and colleagues in the European eel and named *gnrhr1a*, *gnrhr1b* and *gnrhr2* (56). During experimental sexual maturation induced by gonadotropic treatments, the expression of pituitary *gnrhr2* (corresponding to *gnrhr2b* in Ciani’s nomenclature) largely increases, suggesting a major role of this receptor in the regulation of gonadotroph cells in male and female eels (56). The increase in eel *gnrhr2* expression may likely result from a positive feedback by gonadal hormones, the production of which is stimulated during experimental maturation [for review (49)]. Steroid hormones exert positive feedback on brain GnRH and pituitary LH in the eel as shown by our early studies *in A. anguilla* [ (57–62); for review (49)] as well as by investigations in *A. japonica* (63, 64).

We recently investigated the regulation of *gnrhr2* expression in primary cultures of eel pituitary cells and showed inhibitory effects of kisspeptins (65), neurokinin B (66) and gonadotropin-inhibitory hormone (67). In the present study, we investigated the effects of sex steroids, corticosteroid, as well as of activins, on the expression of *gnrhr2* by European eel pituitary cells *in vitro*. In order to get more insights on the regulation of eel *gnrhr2* expression, we performed *in silico* analysis of the *gnrhr2* proximal promoter in the European eel to infer the presence of potential transcription factor binding sites. Comparative analyses were made with the *gnrhr2* promoters of the Japanese eel (*Anguilla japonica*) and other teleost species (zebrafish *Danio rerio* and medaka *Oryzias latipes*), as well as with the *gnrhr1* promoters of two mammals (human *Homo sapiens* and mouse *Mus musculus*). While investigations on *gnrhr* promoters are still lacking outside mammals, the present study allowed us to raise the first data on response elements potentially involved in the regulation of *gnrhr* expression in teleosts. Comparison with *gnrhr* promoters in mammals led us to infer some evolutionary conserved features across vertebrates.

## 2 Materials and methods

### 2.1 Animals

Freshwater female European eels were at the prepubertal “silver” stage, which corresponds to the last continental phase of the eel life cycle, preceding the oceanic reproductive migration. Cloning, tissue distribution and primary cultures were performed using female silver eels purchased from Gebr. Dil import-export BV (Akersloot, The Netherlands) and transferred to MNHN, France. Animals were anesthetized by cooling and then killed by decapitation under the supervision of authorized person (KR, N°R-75UPMC-F1-08) according to the protocol approved by French Cuvier Ethic Committee (N°68-027). Pituitaries were collected in cell serum-free culture medium (CM: Medium 199 with Earle’s salt and sodium bicarbonate buffer, 100 U/ml penicillin, 100 µg/ml streptomycin, 250 ng/ml fungizone; Gibco, Thermo Fisher Scientific, Illkirch, France) just prior to dispersion (15 to 20 eel pituitaries per cell culture).

### 2.2 Hormones and chemicals

Sex steroids (estradiol, E2; testosterone, T; 11-ketotestosterone, 11-KT; progesterone, P), cortisol (F), and aromatase inhibitor, fadrozole, were all purchased from Sigma-Aldrich (Saint-Quentin Fallavier, France). Recombinant human/mouse/rat activins A and B were purchased from R and D Systems (Lille, France). The recombinant human/mouse/rat activins used in our study have high amino-acid identity with the eel ones, and we previously showed that they were effective in stimulating *fshβ* expression by primary cultures of European eel pituitary cells (68). Sex steroids and cortisol were dissolved in ultrapure ethanol (Sigma-Aldrich), activins A and B in sterile calcium-free phosphate-buffered saline (PBS) (Gibco), and fadrozole in dimethyl sulfoxide (DMSO, Sigma-Aldrich) to prepare stock solutions that were stored at -20 °C.

### 2.3 Primary cultures of eel pituitary cells

Dispersion and primary cultures of pituitary cells were performed using an enzymatic and mechanical method as previously described (69). Briefly, pituitaries from 15 to 20 eels per experiment were incubated at 25 °C in a solution of porcine type II trypsin (Sigma-Aldrich) in PBS. After 1h, the trypsin solution was replaced by a solution of DNase (Sigma-Aldrich) and soya bean trypsin inhibitor (Sigma-Aldrich) for 30 min. Pituitaries were cut in 1mm slices using a McIlwain Tissue Chopper (Thermo Fisher Scientific), and then washed with PBS and mechanically dispersed by repeated passages through a plastic transfer pipette (Falcon, Thermo Fisher scientific, Illkirch, France). After estimating the number of viable cells by Trypan Blue exclusion (Sigma-Aldrich), cells were plated on 96-well plates (62,500 cells/well) pre-coated with poly-L-lysine (Sigma-Aldrich). Cultures were performed in culture medium (CM) at 18 °C under 3% CO _2_ and saturated humidity.

Treatments were started 24 h after the beginning of culture to allow cell attachment (Day 0). Replicates of 5 wells for control and each treated group were used. Stock solutions were diluted in CM just before addition to the culture wells. The final concentration of ethanol or DMSO in culture wells never exceeded 0.2%; control wells were treated with the same concentration of ethanol and/or DMSO in CM. Culture medium was changed and treatment added to the cells on Day 0, Day 3, and Day 7. Cultures were stopped on Day 8. The effects of treatments were tested in three independent experiments performed on different cell preparations from different batches of eels. Similar responses were observed in the independent experiments.

Total RNA was directly extracted in wells using the Cell-to-cDNA II Kit (Ambion, Thermo Fisher scientific, Illkirch, France) according to the manufacturer’s recommendations. Cells were washed with PBS and lysed with Cell Lysis II Buffer (80 µl/well). The lysates were digested with RNAse-free DNase I (Roche, Thermo Fisher scientific, Illkirch, France). Four µl of RNA solution of each sample was then reverse transcribed with a SuperScript III First Strand cDNA Synthesis Kit (Invitrogen, Thermo Fisher scientific, Illkirch, France). The samples obtained were stored at –20 °C until qPCR.

### 2.4 Real-time quantitative PCR

Gene specific primers were previously designed based on the nucleotide sequence of the European eel *gnrhr2* (56)] and *β-actin* (70) cDNA, the latter being used as reference gene. Basal *gnrhr1a* and *gnrhr1b* expressions were below the threshold of detection in primary cultures of eel pituitary cells (65–67), and none of the treatments tested in the present study could induce their expression above this limit. For this reason, qPCR data were not reported for these two genes.

qPCRs were prepared with 4 µl of diluted cDNA template, 2 µl of PCR grade water, 2 µl of SYBR Green master mix and 1 µl of each forward and reverse primer (500 nM each at final concentration). The protocol was as previously described for *β-actin* (70) and for *gnrhr2* (65, 66). Serial dilutions of cDNA pools of pituitary cells were used as a standard curve. One chosen dilution was also included in each run as a calibrator. Each qPCR run contained a non-template control (cDNA substituted by water) for each primer pairs to confirm that reagents were not contaminated. The specificity of each reaction was assessed by melting curve analysis to ensure the presence of only one product. Each sample was analyzed in duplicate by qPCR. Normalization of data was performed using *β-actin* mRNA levels.

### 2.5 Statistics

Results of qPCR are given as mean ± SEM. Means were compared by one-way ANOVA Tukey’s multiple comparison test using Instat (GraphPad Software Inc., San Diego, Calif., USA).

### 2.6 *In silico* retrieval of eel *gnrhr2* gene sequences for promoter analysis

To investigate the upstream regulatory regions of the *gnrhr2* gene, genomic sequences were retrieved from both the European eel and the Japanese eel genomes. The genome assembly for the European eel was obtained from GenBank (GCA_013347855.1), and the *gnrhr2* gene was identified on chromosome 16 (accession number: NC_049216). For the Japanese eel, sequence data were collected from genome assembly GCA_025169545.1, with the *gnrhr2* gene found to be similarly located on chromosome 16 (accession number: CM_045898). The 5’-flanking regions upstream of the coding sequence (up to about 2 Kb) were extracted for promoter analysis.

### 2.7 Prediction of transcription factor-binding sites in eel *gnrhr2* promoter

Promoter analyses were subsequently performed on the retrieved 5’-flanking sequences to identify putative transcription factor-binding sites that may contribute to the regulation of *gnrhr2* gene expression. Predictions were made using the PROMO tool, which is based on the TRANSFAC database, as well as the JASPAR database (https://jaspar.elixir.no) (71). These tools were supplemented by information from previously published studies (72–77).

Several potential regulatory elements were identified based on sequence similarity to motifs previously reported in mouse [for review (77)]: Activating Protein 1 (AP1); cAMP Response Element (CRE); Downstream Activin Regulatory Element (DARE); GnRH Receptor Activating Sequence (GRAS); LIM/homeobox protein LHX3 binding site (LHX3); Steroidogenic Factor 1 (SF1); Sequence Underlying Responsiveness to GnRH (SURG1 and SURG2).

### 2.8 Comparison with transcription factor-binding sites in the promoters of other teleost *gnrhr2* and mammalian *gnrhr1*

To assess the conservation and divergence of transcriptional regulatory elements across species, we compared the European and Japanese eel *gnrhr2* promoter regions with *gnrhr* promoter sequences from selected vertebrate species. The coding sequence of European eel *gnrhr2* was used as a query to perform a translated BLAST (tBLASTn) search against the NCBI nucleotide and genome databases for identification of orthologous genes in other teleosts. The closest sequences retrieved by blasting were in zebrafish, the *gnrhr4* gene (GeneID: 100001586) located on chromosome 18 (NC_007129), and in medaka, the *gnrhr4* gene (GeneID: 100125529) located on chromosome 6 (NC_019864). The close relationship between eel *gnrh2* and these genes in zebrafish and medaka, shown in our study by blasting, is in agreement with Ciani’s phylogeny which clusters them all in the *gnrhr2b* subtype (39). We also compared to the promoter of the mammalian *gnrhr* expressed in the pituitary, using human and mice *gnrhr1* promoters. The human *gnrhr1* (Gene ID: 2798) and mouse *gnrhr1* (Gene ID: 14715) are located on chromosome 4 (NC_000004) and chromosome 5 (NC_000071), respectively. For promoter analysis in teleosts and mammals, the *gnrhr* genomic regions were retrieved to extract up to ~2.0 kb upstream sequences from the ATG start codon, which were analyzed for putative transcription factor-binding sites. Previously published mammalian *gnrh1* promoter sequences and response elements [for review (77)] were also used.

## 3 Results

### 3.1 Effects of steroid hormones on *gnrhr2* transcript levels in eel pituitary cells *in vitro*

**Figure 1:**
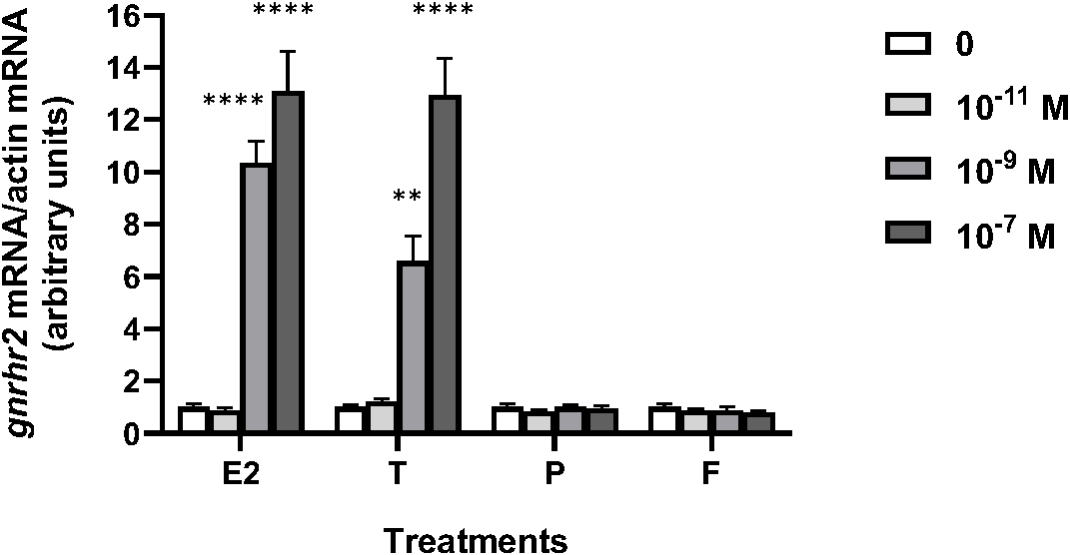
Effects of steroid hormones on *gonadotropin-releasing hormone receptor 2* (*gnrhr2)* transcript levels in primary cultures of eel pituitary cells. Eel pituitary cells were treated with various concentrations of estradiol (E2), testosterone (T), progesterone (P) or cortisol (F) for 8 days. The mRNA levels of *gnrhr2* were quantified by qPCR. Data were normalized against *β-actin*. The Figure displays the results from a representative experiment of three independent cell culture experiments. Mean ± SEM; n=5 well replicates. **, *P* < 0.01 and ****, *P* < 0.0001 *versus* controls, ANOVA.

Various concentrations (from 10^–11^ to 10^–7^ M) of sex steroids E2, T, P, as well as glucocorticoid, F, were tested over 8 days of culture according to previous experiments (70) (**Figure 1**). E2 had no effect at 10^–11^ M but significantly increased *gnrhr2* mRNA levels at 10^–9^ and 10^–7^ M (x10 and x13, as compared to controls, respectively; *P* < 0.0001). T had no effect at 10^–11^ M but significantly increased *gnrhr2* mRNA levels at 10^–9^ and 10^–7^ M (x6.6, *P* < 0.01 and x13, *P* < 0.0001, respectively). No effect of P nor F was observed at the three concentrations tested.

### 3.2 Effect of an anti-aromatase, fadrozole, on sex steroid stimulation of *gnrhr2* transcript levels in eel pituitary cells *in vitro*

**Figure 2:**
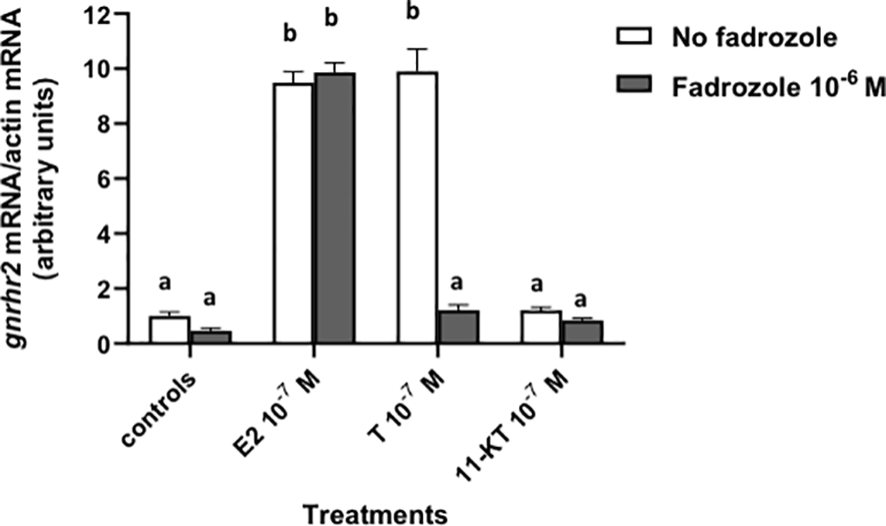
Effect of fadrozole, an aromatase inhibitor, on sex steroid effects on *gonadotropin-releasing hormone receptor 2 (gnrhr2)* transcript levels in primary cultures of eel pituitary cells. Eel pituitary cells were treated with 10^–7^ M of estradiol (E2), testosterone (T) or 11-ketotestosterone (11-KT) in the presence or not of 10^–6^ M fadrozole for 8 days. The mRNA levels of *gnrhr2* were quantified by qPCR. Data were normalized against *β-actin*. The Figure displays the results from a representative experiment of three independent cell culture experiments. Mean ± SEM; n=5 well replicates. Different letters indicate significant differences (*P* < 0.0001) between groups, ANOVA.

Fadrozole (10^–6^ M), an inhibitor of aromatase, was tested alone or in the presence of 10^–7^ M sex steroids, E2, T or 11-KT (a non-aromatizable androgen) (**Figure 2**). Fadrozole had no effect alone and did not affect the stimulatory effect of 10^–7^ M E2 on *gnrhr2* mRNA levels (x9.9 in the presence of fadrozole as compared to controls, *versus* x9.5 in the absence of fadrozole). In contrast, 10^–7^ M T stimulatory effect (x9.9) on *gnrhr2* mRNA levels was completely suppressed by fadrozole (*P* < 0.0001), reaching control levels. No significant effect of 11-KT on *gnrhr2* mRNA levels at 10^–7^ M was observed in the absence and in the presence of fadrozole.

### 3.3 Effect of activins A and B on *gnrhr2* transcript levels in eel pituitary cells *in vitro*

**Figure 3:**
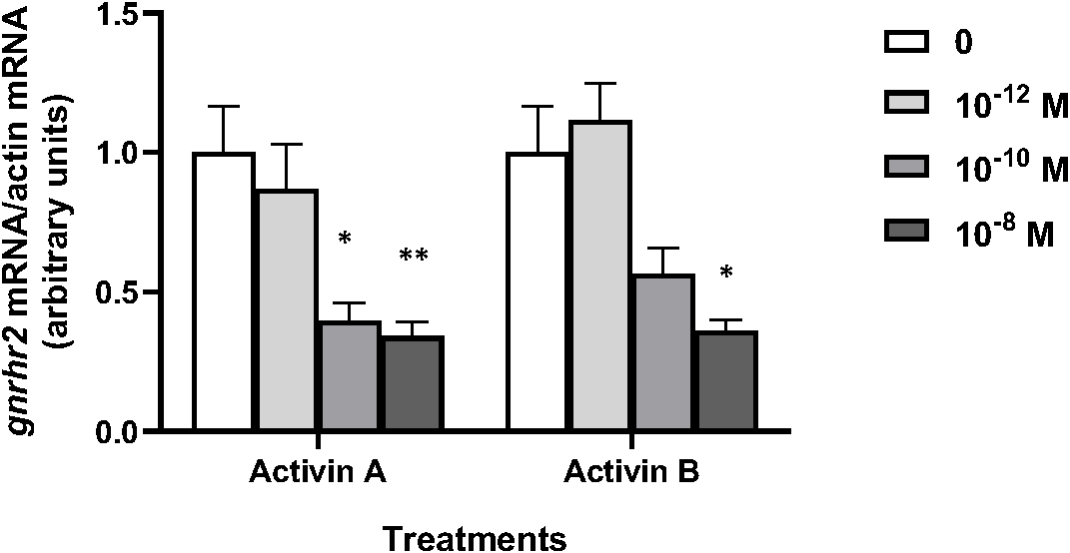
Effects of activins A and B on *gonadotropin-releasing hormone receptor 2 (gnrhr2)* transcript levels in primary cultures of eel pituitary cells. Eel pituitary cells were treated with various concentrations of activin A or activin B for 8 days. The mRNA levels of *gnrhr2* were quantified by qPCR. Data were normalized against *β-actin*. The Figure displays the results from a representative experiment of three independent cell culture experiments. Mean ± SEM; n=5 well replicates. *, *P* < 0.05 and **, *P* < 0.01 *versus* controls, ANOVA.

Various concentrations of peptide hormones activin A and activin B (from 10^–12^ to 10^–8^ M) were tested over 8 days of culture according to previous experiments (68) (**Figure 3**). Both hormones had no effect at 10^–12^ M, but *gnrhr2* mRNA levels were significantly decreased by activin A at 10^–10^ and 10^–8^ M (x0.4, *P* < 0.05 and x0.34, *P* < 0.01 as compared to controls, respectively), and by activin B at 10^–8^ M (x0.36; *P* < 0.05, as compared to controls).

### 3.4 Genomic structure of eel *gnrhr2* gene

**Figure 4:**
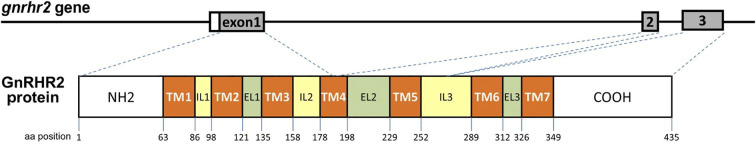
Schematic representation of the European eel *gonadotropin-releasing hormone receptor 2 (gnrhr2)* gene and its encoded protein structure. The European eel *gnrhr2* gene, retrieved from genome assembly GCA_013347855.1, spans 9,860 bp on chromosome 16 (NC_049216) and consists of three exons and two introns. The main transcript (XM_035396496) encodes a 435-amino acid GnRHR2 protein (XP_035252387). The gene structure is shown in the upper panel, with exon numbers indicated. The corresponding GnRHR2 protein structure is illustrated below, showing the seven transmembrane domains (TM1–TM7), the three extracellular loops (EL1–EL3), and the three intracellular loops (IL1–IL3). Exon-protein domain correspondence is indicated by dotted lines. Similar genomic structure was found in the Japanese eel, with retrieved *gnrhr2* gene sequence on chromosome 16 (CM_045898) from genome assembly GCA_025169545.1.

The *gnrhr2* gene sequence of the European eel was retrieved from GenBank genome assembly GCA_013347855.1. The *gnrhr2* gene was identified on chromosome 16 (NC_049216), spanning the genomic region from position 12,519,164 to 12,529,023 (Gene ID: 118215598). It is composed of three exons separated by two introns (**Figure 4**), and the entire gene covers 9,860 bp on the chromosome. The CDS of the gene, registered under accession number XM_035396496, is 1,308 bp in length and encodes a protein of 435 aa, with the corresponding protein sequence recorded under accession number XP_035252387. In the Japanese eel, *gnrhr2* was not previously annotated. Gene sequence information was retrieved from GenBank genome assembly GCA_025169545.1, and *gnrhr2* was found on chromosome 16 (CM_045898), between positions 23,834,808 and 23,841,537. We predicted exon-intron boundaries following the GT-AG rule. The Japanese eel *gnrhr2* gene also comprises three exons and two introns (**Figure 4**). Its CDS is 1,308 bp long and encodes a protein of 435 aa, showing a high sequence conservation between these two eel species (96.79% identity).

The *gnrhr2* genes from the European eel and the Japanese eel display a highly conserved exon structure in terms of coding sequence organization and functional domain distribution (**Figure 4**). In the European eel, exon 1 comprises 582 nucleotides, encoding the first three transmembrane (TM) domains and a portion of the fourth TM domain. Exon 2 comprises 205 nucleotides, encompassing the remaining part of the fourth TM domain and the entire fifth TM domain. Exon 3 consists of 521 nucleotides, encoding the sixth and seventh TM domains along with the remainder of the coding sequence. Similarly, in the Japanese eel, exon 1 contains 582 nucleotides and encodes the same TM domains as in the European eel. Exon 2 includes 204 nucleotides, covering the rest of the fourth TM and the fifth TM domain. Exon 3 spans 522 nucleotides, responsible for encoding the sixth and seventh TM domains and the remaining coding sequence. These findings highlight the strong conservation in the structural organization of the *gnrhr2* gene between the two eel species.

### 3.5 Analysis of eel *gnrhr2* promoter region

**Figure 5:**
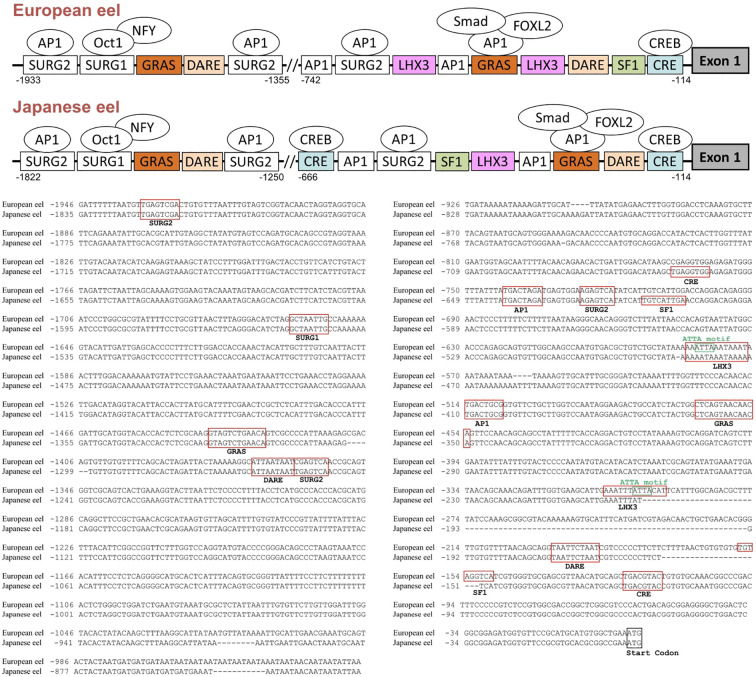
Predicted transcription factor binding sites in the 5′-flanking region of the European and Japanese eel *gonadotropin-releasing hormone receptor 2 (gnrhr2)* genes. Promoter analysis of the *gnrhr2* gene revealed multiple putative transcription factor-binding sites within ~2.0 kb upstream of the ATG start codon. A schematic representation of the *gnrhr2* promoter response elements, with some potential binding factors, in both eel species, is provided in the upper panel. The exon 1 coding region is depicted as a dark grey box on the right. The alignment of *gnrhr2* promoter sequences between European eel and Japanese eel is shown in the lower panel, with identified response elements boxed in red, and the start codon of exon 1 boxed in black. AP1, activating protein 1; CRE, cAMP response element; CREB, CRE binding protein; DARE, downstream activin regulatory element; FOXL2, Forkhead box L2; GRAS, GnRH receptor activating sequence; LHX3, LIM/homeobox protein LHX3 binding site; NFY, Nuclear Factor Y; OCT1, Octamer binding transcription factor 1; SF1, steroidogenic factor 1; SMAD, Suppressor of mothers against decapentaplegic; SURG, sequence underlying responsiveness to GnRH.

The predicted response elements are indicated in the promoter sequences of the European eel and the Japanese eel *gnrhr2* genes (**Figure 5**). Sequence analysis up to ~2.0 kb upstream from the ATG start codon in the European eel revealed multiple transcription factor binding sites including a cAMP Response Element (CRE) at position nt -114, that binds CRE-binding protein (CREB), along with a putative binding site for Steroidogenic Factor 1 (SF1) at nt -149. Two TAAT/ATTA motifs were located at nt -187 and nt -1362, representing potential Downstream Activin Regulatory Element (DARE) homeodomain protein binding sites. The binding sites for LHX3, a LIM-homeodomain transcription factor, were found at nt -293 and -572. Two GnRH Receptor Activating Sequence (GRAS) elements known to mediate Suppressor of Mothers Against Decapentaplegic (SMAD) and Forkhead box L2 (FOXL2) binding in mammals, were identified at nt -454 and -1428, and two binding sites for Activating Protein 1 (AP1) were observed at nt -507 and -735. In addition, three Sequence Underlying Responsiveness to GnRH (SURG) – 2 elements (SURG2) were detected at nt -720, -1355, and -1926, which are also AP1 binding sites. A SURG1, an element that was previously reported as interacting with transcription factors such as OCTamer binding transcription factor 1 (OCT1 formally named POU2F1) and Nuclear Factor Y (NFY), was present at nt -1655. These elements were also identified in the promoter sequence of the Japanese eel *gnrhr2*: a CRE next to the first exon (at nt -114) as in the European eel, but also an additional CRE at -659; two DARE at nt -165 and -1257, two GRAS at nt-350 and -1317, two AP1 at nt -403 and -634, as in the European eel; a single LHX3 at nt -472 corresponding to the second one of the European eel; a SF1 but located further away from the first exon, at nt -604, as compared to the European eel; three SURG2 at nt -619, -1250 and -1815, and a SURG1 at nt -1544, as in the European eel.

### 3.6 Comparison of transcription binding sites in promoter region of eel *gnrhr2* and other species *gnrhr*

**Figure 6:**
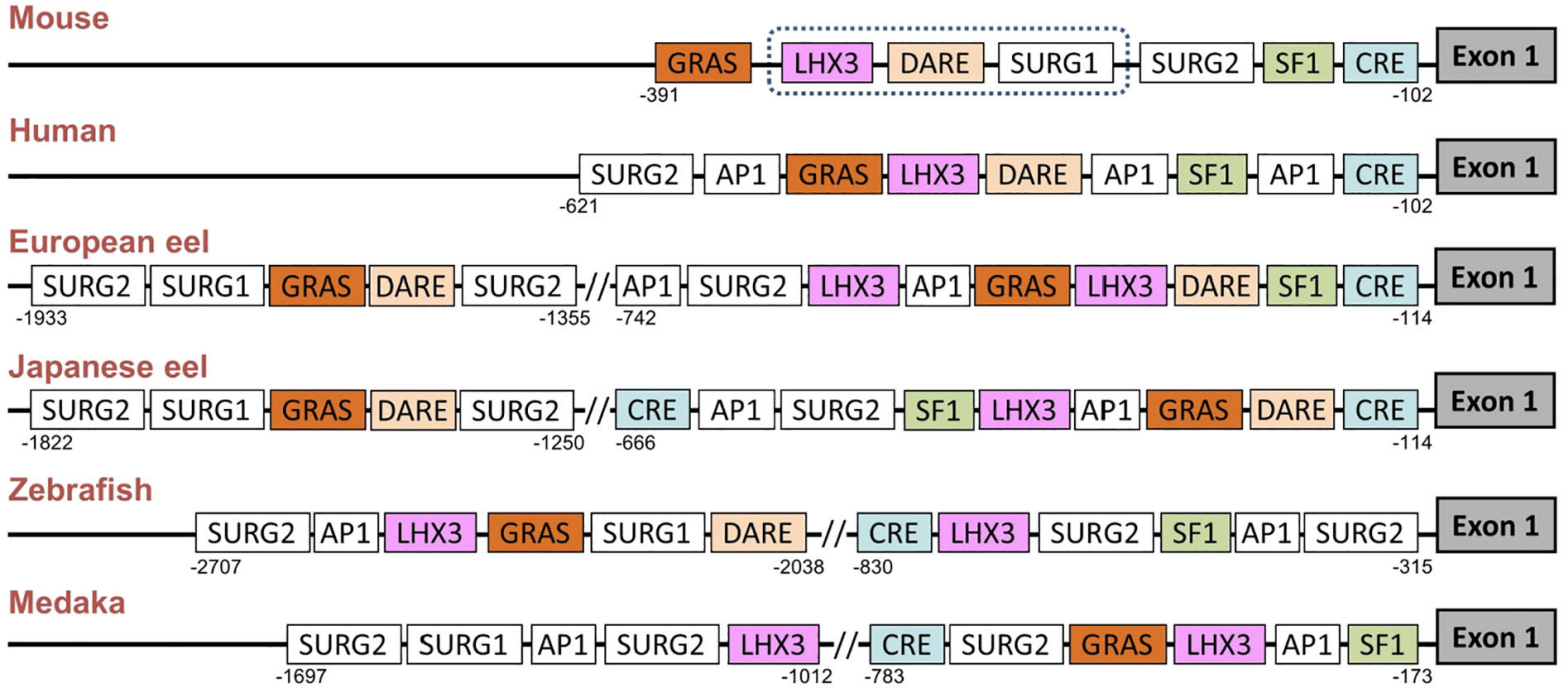
Comparison of predicted transcription factor binding sites in *gonadotropin-releasing hormone receptor (gnrhr)* promoters between European and Japanese eels and other teleost and mammals. The figure displays a schematic representation of promoter response elements of *gnrhr1* from mouse and human and of *gnrhr2* from European eel, Japanese eel, zebrafish and medaka. For gene references, see Materials and Methods. The exon 1 coding region is depicted as a dark grey box on the right. The dotted line in the mouse promoter indicates a region with several binding elements overlapping. AP1, activating protein 1; CRE, cAMP response element; DARE, downstream activin regulatory element; GRAS, GnRH receptor activating sequence; LHX3, LIM/homeobox protein LHX3 binding site; SF1, steroidogenic factor 1; SURG, sequence underlying responsiveness to GnRH.

**Figure 7:**
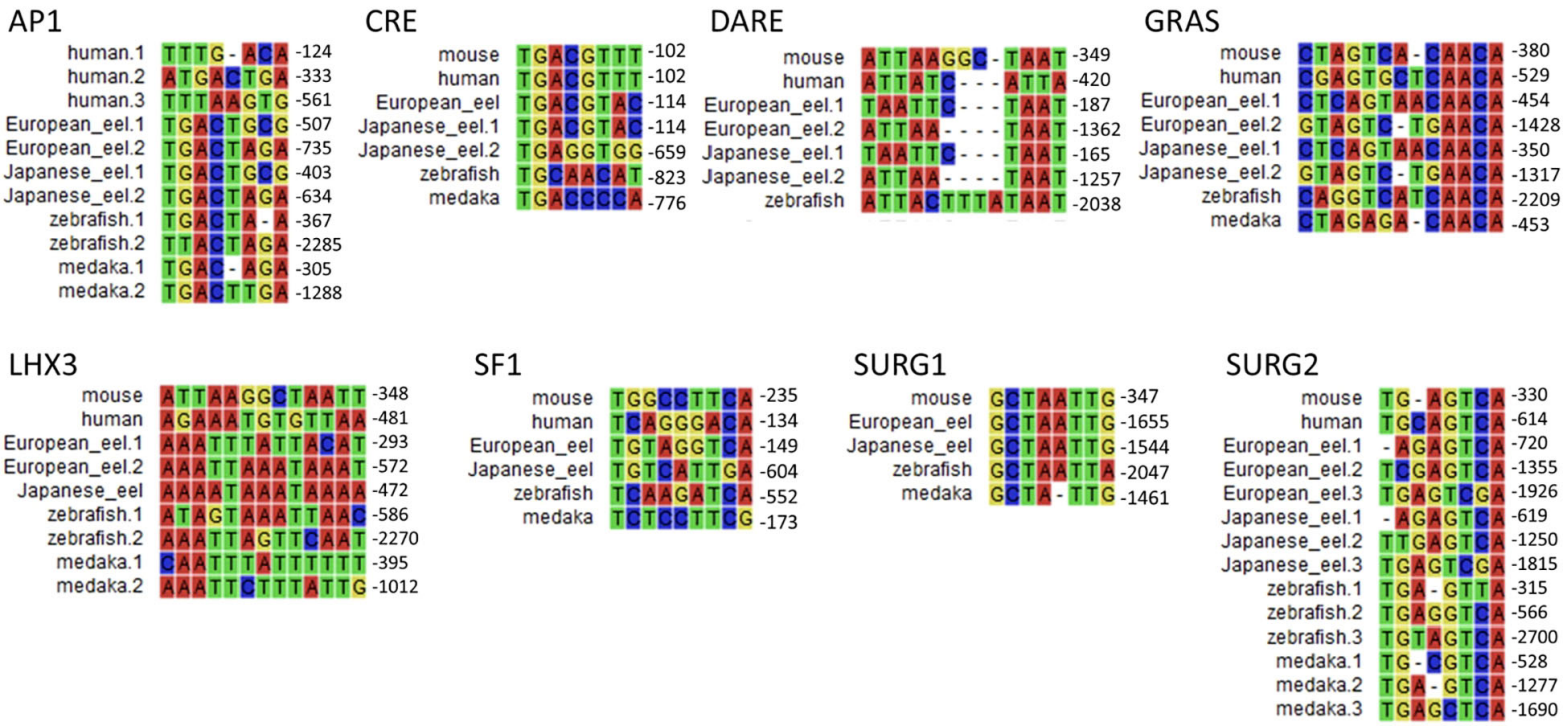
Comparison of predicted transcription factor binding motif sequences in *gonadotropin-releasing hormone receptor (gnrhr)* promoters between European and Japanese eels and other teleost and mammals. Multiple sequence alignments of predicted response elements were performed using promoter sequences of *gnrhr1* from mouse, and human, and *gnrhr2* from European eel, Japanese eel, zebrafish, and medaka. Each panel displays a typical response element. When a response element is present in multiple locations in a promoter, the numbers following species names (e.g., European eel.1, Japanese eel.2) correspond to the relative positions, upstream of the ATG start codon. AP1, activating protein 1; CRE, cAMP response element; DARE, downstream activin regulatory element; GRAS, GnRH receptor activating sequence; LHX3, LIM/homeobox protein LHX3 binding site; SF1, steroidogenic factor 1; SURG, sequence underlying responsiveness to GnRH.

The promoter regions of *gnrhr2* from the European eel and the Japanese eel were compared to those of *gnrhr2* from other teleosts (zebrafish and medaka) and of *gnrhr1* from human and mouse. The distribution of consensus transcription factor binding elements identified in these promoter regions are schematically illustrated in **Figure 6**, including CRE, SF1, DARE, LHX3, GRAS, AP1, as well as SURG1 and SURG2. The sequence alignments of predicted response elements within the *gnrhr* promoter regions among selected species, with their relative positions, are presented in **Figure 7**.

Among these predicted response elements, a CRE site was found in each species examined, with an additional one in the Japanese eel. The CRE was located just upstream of exon 1 in the two eel species, as in mammals, while it was positioned more distantly in zebrafish and medaka, at a location corresponding to that of the second CRE of the Japanese eel. A SF1 was retrieved in the promoter of each species investigated.

In the mouse promoter, several binding elements overlap (SURG1, DARE and LHX3), while we found them separated in other species. SURG1 and SURG2, which were originally identified in *gnrhr* promoter in mammals, were also detected in teleost species. A single SURG1 element was located in all analyzed teleost promoters, whereas multiple SURG2 elements were found. We retrieved DARE in the proximal region of the promoter in human as well as in the European and Japanese eels; an additional DARE was also found in a more distant region of the promoter in both eel species; in zebrafish, a DARE was at a position possibly corresponding to the second DARE of the eels, while no DARE was retrieved in medaka. Concerning LHX3, it was present in all species, with an additional one in teleosts (European eel, zebrafish and medaka).

As previously shown in the mouse *gnrhr1* promoter, the GRAS motif was consistently detected in all examined species, with two GRAS found in both eels. The single GRAS motif found in the proximal region of the promoter in medaka may correspond to the first one of the eels, while the one found more distantly in the zebrafish may correspond to the second one of the eels. Repeated AP1 motifs appeared frequently across species, with two sites observed in all analyzed teleosts, and three sites in human.

## 4 Discussion

### 4.1 High conservation of *gnrhr* promoter response elements between teleosts and mammals

The response elements characterized in the eel *gnrhr2* promoters are similar to the major ones involved in the tissue-specific activity, and in the response to regulatory factors, for mammalian *gnrhr1* promoters in the pituitary gonadotroph cells [ (78–80); for review (77)]. We also identified them in the *gnrhr2* promoter of two other teleost model species, zebrafish and medaka. This suggests a high evolutionary conservation of the molecular mechanisms underlying *gnrhr* gene expression in gonadotroph cells, even between distant species like teleosts and mammals. These shared transcription factor binding motifs include CRE, SF1, DARE, LHX3, GRAS, AP1, SURG1 and SURG2, which have been shown to mediate either basal or regulated expression of *gnrh1* in mammal species. Notably, in rodents, mouse and rat, SF1 in the proximal promoter region is essential for *gnrhr1* gonadotroph-specific expression and basal transcriptional activity (78, 80). This same factor was also identified in the eel and other teleost *gnrhr2* promoters, suggesting conserved pituitary-specific regulation. Various homeobox factor binding sites, which participate in the gonadotroph-specific expression of the mammalian *gnrhr1* promoter, are also found in the eel and other teleost *gnrhr2*, such as SURG1, which binds OCT1 and NFY, both involved in directing basal expression and GnRH-stimulated expression on the *gnrhr* gene (75). *In vivo* chromatin immunoprecipitation (ChIP) assays confirmed that OCT1 and NFY bind to the SURG1 element in the mouse, and this binding increases in response to GnRH stimulation (75). In the mouse, SURG2 overlaps with a conserved AP1 consensus binding site and the AP1 binding site is essential for *gnrhr* gene expression under GnRH stimulation, while GRAS is a composite regulatory element whose functional activity depends on the binding of Smad proteins, AP1, and FOXL2 and mediates both activin and GnRH responsiveness (75). We identified a GRAS motif in the *gnrhr* promoters of all analyzed species, suggesting a potentially conserved regulatory mechanism across vertebrates. LHX3 binding sites, which directly activate the mouse *gnrhr1* promoter through an ATTA core motif (76, 81), were also found in the eel and other teleost *gnrhr2* promoters. The DARE motif contains TAAT/ATTA motifs, which were shown to bind homeodomain transcription factors such as LHX3 in the mouse *gnrhr1* promoter (82). DARE was also identified in the present study in the promoters of *gnrhr1* in human, and *gnrhr2* in European eel, Japanese eel, and zebrafish, indicating a conserved regulatory site across vertebrates.

In the present study, the identification of response elements in *gnrhr1* promoter of mammalian species (human and mouse) and in *gnrhr2* promoter of teleost species (eels, zebrafish and medaka) show a conservation of key regulatory elements in the *gnrhr* promoter in the osteichthyan lineage, which encompass actinopterygians (such as teleosts) and sarcopterygians (such as mammals). This reveals an ancient origin and evolutionary conservation of transcriptional control mechanisms governing *gnrhr* expression by pituitary gonadotroph cells. Furthermore, the identification of these conserved elements in eel supports this basal teleost as a valuable comparative model for understanding the evolution of vertebrate reproductive endocrinology. Further comparative analyses in other lineages such as chondrichthyans (cartilaginous fishes) and cyclostomes (jawless vertebrates) would allow the elucidation to whether this regulatory system is already present in early vertebrates, before the emergence of jawed vertebrates.

### 4.2 *In vitro* and *in silico* insights for estrogen-specific stimulation of eel pituitary *gnrhr2* expression

In our present study, we showed a stimulatory effect of E2, as well as of T, on *gnrhr2* mRNA levels in primary culture of eel pituitary cells. This effect was estrogen-specific, as no such a stimulatory effect was induced by a non-aromatizable androgen 11-KT and the stimulatory effect of T was abolished in the presence of an aromatase inhibitor, fadrozole.

In various teleost species, sex steroids also modulate pituitary responsiveness to GnRH and regulate *gnrhr* mRNA levels. A study in primary cultured pituitary cells also reported an increase in pituitary *gnrhr3* but not *gnrhr1* mRNA levels in tilapia after E2 exposure while T was not tested (41). In the black porgy, after both E2 and T treatments, *gnrhr1* (*gnrhr2bb*) mRNA levels were increased in dispersed pituitary cells, while 11-KT did not change them, suggesting that in this species like in the eel, the stimulatory effect of T on *gnrhr* expression may be mediated by aromatization, thus being estrogen-specific (47). Another *in vitro* study in Atlantic cod compared the effects of E2, T and dihydrotestosterone DHT [a non-aromatisable androgen but whose metabolite 3β-diol, also named 5α-androstane-3β, 17β-diol, binds to estrogen receptor β (83, 84)] and demonstrated that all increased pituitary *gnrhr2a* mRNA levels, without affecting *gnrhr1b* (85). A recent study, using *ex vivo* whole pituitaries of Atlantic salmon, reported that pituitary *gnrhr2bba* expression is stimulated by both E2 and 11-KT, indicating both estrogenic and androgenic effects (86).

*In vivo* studies in teleosts also reported that E2 treatment induces an increase in pituitary *gnrhr* mRNA levels. In tilapia, both *gnrhr1* and *gnrhr3* mRNA levels increase after E2 treatment (41), while in black porgy, only *gnrhr1* (*gnrhr2bb*) increases (47, 87). The effect of T was tested in Atlantic salmon, showing a stimulatory effect on the pituitary transcripts of *gnrhr4* (45). In contrast, in their study on endocrine disrupting chemicals in the hermaphroditic fish *Kryptolebias marmoratus*, Rhee and colleagues noted that exposure to E2 in water induces a decrease in *gnrhr* mRNA levels (88).

In rat, E2 treatment of adult female pituitary cells induces an increase in GnRHR number as well as on GnRH-induced LH release (89–91). In ewe, E2 is also able to increase GnRHR number (92) and *gnrhr1* mRNA levels by primary cultures of pituitary cells (93, 94). In contrast, E2 has no effect on *gnrhr1* mRNA levels expressed in mouse gonadotrope cell line, LβT2 (95).

The positive *in vitro* effect of E2 or of T after aromatization on *gnrhr2* transcripts in the European eel supports our previous *in vivo* data showing the need to use sex steroids to sensitize the eel pituitary LH response to GnRH (53). This pathway is likely involved in the increase of *gnrhr2* expression observed during eel sexual maturation (56) via the positive feedback of sex steroids.

Despite the strong estradiol-specific regulation of eel *gnrhr2*, we did not evidence a typical estrogen response element (ERE) within the eel *gnrhr2* proximal promoter region that we have investigated. This aligns with findings in mammals, in which canonical ERE could not be identified in the *gnrhr1* promoter, and thus would not mediate E2 regulatory effect on *gnrhr1* expression. Instead, non-classical pathways appear to mediate E2 action on *gnrhr1* transcription across mammalian species and cell types [ (20); for reviews (77, 96)]. In human ovarian (OVCAR3) and breast (MCF7) cell lines, it was shown that E2-activated ERα represses *gnrhr1* gene transcription via an indirect mechanism involving CBP (CREB binding protein) and AP1 (73). A similar mediation by CREB was also demonstrated to underlie the stimulatory effect of E2 on *gnrhr* expression by ovine pituitary cells (20). We identified CRE and AP1 in the eel *gnrhr2* promoter, as well as in other teleost *gnrhr2* promoters, both response elements implicated in E2 signaling in mammalian *gnrhr1* promoters. We suggest that the stimulatory effect of E2 on *gnrhr2* expression, observed in our study, may be mediated via these two response elements. These findings on estrogen-specific stimulation of *gnrhr2* expression levels in eel pituitary cells contribute significantly to the understanding of the conservation of the regulation of *gnrhr* by estrogens across vertebrates.

The increase in *gnrhr* expression further highlights the multiple targets of the steroid positive feedback on brain-pituitary gonadotropic axis in the eel, together with the previous demonstration of the stimulatory effects of gonadal steroids on the synthesis of brain GnRH and pituitary LH. We suggest that the positive regulation by E2 of *gnrhr2* expression is exerted on LH cells. Differently from the situation in mammals and other tetrapods, where both gonadotropins are produced by the same pituitary cells, LH and FSH are expressed by distinct pituitary cells in teleosts, including in the eel as shown by *in situ* hybridization (ISH) (97). Furthermore, a recent study proposed a dual neuroendocrine control of gonadotropins in teleosts, with GnRH acting as LH releasing hormone while cholecystokinin as FSH releasing hormone (98). Future experiments, such as double ISH or ISH coupled to immunohistochemistry on dispersed pituitary cells, an approach already set up for DA receptors in the European eel (99), could investigate which pituitary gonadotroph cell(s) express *gnrhr2*.

### 4.3 Lack of evidence for progesterone and cortisol regulation of eel pituitary *gnrhr2* expression

In the European eel, we observed no direct effect of progesterone on *gnrhr2* expression *in vitro*. To our knowledge, the unique other study regarding the effect of progestogens on *gnrhr* expression in teleosts was performed in tilapia (41). The authors demonstrated that a progestin, 17α, 20β-dihydroxy-4-pregnen-3-one (DHP), could positively regulate *gnrhr1* and *gnrhr3* mRNA levels by primary cultures of pituitary cells, while only those of *gnrhr1* are elevated *in vivo*. In contrast, in ewe, an inhibitory effect of progesterone on basal or E2-induced *gnrhr1* expression by primary culture of pituitary cells was reported *in vitro* (92, 94, 100). *In vivo*, progesterone had no effect on the basal number and/or mRNA levels of pituitary GnRHR of ovariectomized ewes (18, 101) and cows (102), but reduced their E2-induced number [ewes (101); cows (102)]. In contrast, combined treatment of hypogonadic (*hpg*) female mice with GnRH, E2 and progesterone elevated the pituitary GnRHR number to the same levels as normal mice (103) supporting a positive synergistic effect of progesterone. Further studies may investigate hormone interactions in the regulation of eel *gnrhr2* transcript levels.

A functional progesterone response element (PRE) has been characterized in human *gnrhr1* promoter, which mediates the inhibitory effect of progesterone in human gonadotroph cells via progesterone receptor isoforms PR-A and PR-B (104). Such a PRE binding site has not been found in the eel *gnrhr2* promoter, possibly explaining the absence of progesterone effect on *gnrhr2* transcript levels. It should be noted, however, that other signaling mechanisms may mediate the regulatory effects of progesterone, as no PRE were identified in *gnrhr1* promoter of some mammalian species such as rodents [for review (77)]. Overall, these findings reveal some species-specific patterns in the hormonal regulation of *gnrhr* genes, and indicate the importance of promoter structure in hormone responsiveness.

In our study, cortisol did not induce any change in mRNA levels of pituitary *gnrhr2* in eel pituitary cells. In contrast, one other recent *in vitro* study in a teleost, the Atlantic cod, reports an induction of *gnrhr2a*, but not *gnrhr1b*, by cortisol (85). In mammals, cortisol has no effect on the basal number of GnRHR and/or *gnrhr1* mRNA levels *in vitro* in ewe (105) and *in vivo* in castrated sheep (106, 107). However, *in vivo* it can reduce the stimulatory effect of E2 on the number and mRNA levels of GnRHR in castrated sheep (106, 107), while it increases the stimulatory effect of GnRH on GnRHR number in intact male rats (108). Treatment of mouse gonadotrope cell line, LβT2, with dexamethasone can increase *gnrhr1* mRNA levels only in combination with E2 (95). As stress may largely affect fish reproduction, future studies may further address the interaction between corticosteroids, sex steroid and GnRH in the regulation of GnRHR expression in teleosts.

Cortisol response elements, typically referred to as glucocorticoid response elements (GREs), are essential DNA sequences that mediate the transcriptional effects of glucocorticoids via the direct binding of the glucocorticoid receptor (GR). We did not identify a classical GRE in the promoter of eels nor other teleost *gnrhr2* genes, in line with the current data in mammals. The lack of GREs in the *gnrhr1* promoter across mammalian species suggested more complex mechanisms for glucocorticoid actions. Thus, in the mouse, whose *gnrhr1* promoter does not contain a GRE, the transcriptional regulation of the *gnrhr1* gene by glucocorticoid is ensured by the recruitment of GR to the AP1 region of this promoter (109, 110).

### 4.4 *In vitro* and *in silico* insights for activin inhibition of eel pituitary *gnrhr2* expression

In the European eel, we previously showed that activins oppositely regulate *in vitro fshβ* and *lhβ* expression by pituitary cells, with a stimulatory effect on *fshβ* and an inhibitory effect on *lhβ* mRNA levels (68). Besides their production by the gonads, localization of activins has been demonstrated within the pituitary, in various cell types [gonadotrophs in mammals (111); somatotrophs in teleosts (112)], suggesting paracrine/autocrine actions at the pituitary level. Activins are known to stimulate FSH production and release from gonadotrope cells in teleosts as in other vertebrates including mammals (113, 114).

In the present study, we show that both activins A and B are able to downregulate *gnrhr2* mRNA levels by primary cultures of eel pituitary cells. Few data are available in other teleosts. A recent study using *ex vivo* whole pituitaries of Atlantic salmon post-smolts, exposed to stimulatory environmental conditions that promote sexual maturation (continuous light and 16 °C), showed that in immature but not maturing males, activin A stimulates the expression of *gnrhr2bba* (86), the only paralog out of six being stimulated during precocious male parr maturation (39). No effect of activin A on *gnrhr2bba* expression is observed in immature males exposed to non-stimulatory conditions (86).

Early studies showed that activin A stimulates the synthesis rate (as assayed by density shift technique) of GnRHR by rat pituitary cell cultures (33). This stimulatory effect of activin A on GnRHR is exerted at the transcriptional level as demonstrated in the mouse gonadotrope cell line, αT3-1, using *gnrhr1* mRNA assay, run-off experiments, and transfection experiments of *gnrhr1* promoter/luciferase reporter gene (34). In contrast, in the ovariectomized ewes, activin A decreases the number of GnRH-R (as assayed by the binding of a GnRH agonist) by primary cultures of pituitary cells but has no effect on their increase induced by E2 (115). This suggests species-specific variations in the positive or negative effects of activin on GnRHR in mammals, as in teleosts.

Extensive promoter studies in mice have identified GRAS as a critical regulatory element mediating activin-induced transcriptional activation of *gnrhr1* in gonadotrope-derived cell lines such as αT3–1 and LβT2 cells (79, 81). GRAS functions as a composite enhancer, which recruits SMAD2/3/4 and cooperates with factors like AP1, FOXL2, SF-1 to modulate gene expression via overlapping or adjacent binding motifs [ (74, 79, 116); for review (77)]. We identified GRAS elements in *gnrhr* promoters of each mammalian and teleost species analyzed in this study. In the *gnrhr2* promoters of the European and Japanese eels, we found two putative GRAS motifs, including one in the proximal region near to AP1 binding sites, suggesting a conserved activin-responsive promoter structure.

DARE further enhances responsiveness to activin, coupled with GRAS forming a functionally cooperative “activin-responsive unit (ARU)” within the mouse *gnrhr* promoter to drive transcriptional activation (81). DARE contains TAAT/ATTA motifs, which serve as binding sites for homeodomain transcription factors. For example, LHX3 has been shown to bind directly to DARE and activate transcription of mouse *gnrhr1* when overexpressed (81). Additionally, homeodomain proteins such as Msx1 and Dlx3 also interact with TAAT-rich regions to regulate *gnrhr1* transcription during gonadotroph development, with Dlx3 acting as an activator and Msx1 as a repressor, in the mouse (117). We found a DARE in the eel *gnrhr2* promoter, but with “TAAT/ATTA” separated by only 2 bp rather than 4 bp spacing of murine DARE. As for the eel, we observed a 2bp spacer for the DARE motif of the human *gnrhr1* promoter. In Cherrington et al’s study (81), increasing the spacer length between the tandem TAAT/ATTA motifs from 4 to 5 or 10 bp reduced *gnrhr1* promoter activity, but no data are available concerning a shorter space. Whether this shorter spacing affects transcription factor binding or promoter activity remains to be elucidated.

Activin, which we previously showed to stimulate *fshβ*, while inhibiting *lhβ*, in the European eel (68), exerts an inhibitory effect on *gnrhr2* expression by eel pituitary cells. As discussed above for the effect of steroids, the parallel regulation of *lh* and *gnrhr2* expression by activin supports the hypothesis that the expression and regulation of *gnrhr2* concerns mainly LH cells.

## 5 Conclusion and perspectives

This study gives new information on the regulation of eel pituitary *gnrhr2* expression and provides the first insight into the sequence and response elements of *gnrhr2* promoter in teleosts. Our results show that while activins inhibit *gnrhr2* expression, gonadal steroids exert a positive feedback, mediated by estradiol, on pituitary sensitivity to GnRH in the eel. This may account for the increase in pituitary *gnrhr*2 mRNA levels reported in female and male eels experimentally matured under gonadotropic treatments (56). This increase in *gnrhr* expression further highlights the multiple targets of the steroid positive feedback on brain-pituitary gonadotropic axis in the eel, in line with the regulatory mechanisms of the ovulatory LH surge in mammals. Furthermore, the analysis of the eel *gnrh2* promoter sequence suggests the absence of a classical ERE and the involvement of non-classical response elements such as CRE and AP1, similarly to the situation in mammals. This regulation by estradiol of GnRH receptivity would be an ancient and conserved mechanism across vertebrates. Currently, final oocyte maturation and ovulation in female eels, matured after chronic gonadotropic treatments, are induced by the administration of a progestogen, acting directly at the ovarian level (118, 119). The present finding of the increase in eel pituitary sensitivity to GnRH as a result of the estradiol positive feedback, further supports the use of alternative treatments to induce an endogenous ovulatory LH peak, by the administration of GnRH- agonist together with dopamine-antagonist. Future studies should also aim at deciphering upstream regulation of endogenous GnRH release in the eel, such as pheromones and environmental factors. This study in the eel, a basal teleost representative, contributes to raise basic and applied knowledge on the regulation and evolution of pituitary GnRH receptivity in vertebrates.

## Data Availability

The raw data supporting the conclusions of this article will be made available by the authors, without undue reservation.
